# A case report of postoperative VRSA enteritis: Effective management of rifampicin for vancomycin resistant *Staphylococcus aureus* enteritis after esophagectomy and colon reconstruction

**DOI:** 10.1016/j.ijscr.2018.08.038

**Published:** 2018-08-24

**Authors:** Naoya Okada, Takeo Fujita, Jun Kanamori, Ataru Sato, Yasumasa Horikiri, Takuji Sato, Hisashi Fujiwara, Hiroyuki Daiko

**Affiliations:** Department of Esophageal Surgery, National Cancer Center Hospital East, 6-5-1, Kashiwanoha, Kashiwa, Chiba, 277-8577, Japan

**Keywords:** MRSA, methicillin-resistant *Staphylococcus aureus*, WBC, white blood cell, CRP, C-reactive protein, POD, postoperative day, VRSA, vancomycin resistant methicillin-resistant *Staphylococcus aureus*, UICC, Union Internationale Contre le Cancer, TNM, tumor node metastasis, ABK, arbekacin, LZD, linezolid, DAP, daptmycin, Esophagectomy, Colon reconstruction, VRSA, Rifampicin, Enteritis

## Abstract

**Introduction:**

Vancomycin is one of the most widely used antibiotics for the treatment of serious infectious caused by methicillin-resistant *Staphylococcus aureus* (MRSA). However, reduced susceptibility of *S. aureus* to vancomycin has been observed in recent years. We report on a case of vancomycin resistant methicillin-resistant *Staphylococcus aureus* (VRSA) enteritis after colon reconstruction followed by esophagectomy and completion gastrectomy, with extended lymph node dissection for esophageal squamous cell carcinoma.

**Presentation of case:**

A 66-year old male was referred to our hospital for esophageal carcinoma of clinical stage T3 N0 M0, Stage IIA. From the postoperative day 3, the patient reported heavy watery stools on more then 10 occasions and high fever, and was diagnosed with the methicillin-resistant *Staphylococcus aureus* (MRSA) enteritis. We administered vancomycin to treat the enteritis, although a subsequent stool culture indicated VRSA instead of MRSA. Rifampincin treatment was initiated and the patient’s symptoms improved.

**Discussion:**

In this case report, this patient underwent esophagectomy, total resection of the gastric remnant, and colon reconstruction, and it is likely that methicillin-resistant *Staphylococcus aureus* (MRSA) from the upper airway system, which is not exposed to gastric acid, proliferated in the interposed colon and resulted in MRSA enteritis.

**Conclusions:**

Rifampicin represents an effective treatment strategy for postoperative VRSA enteritis.

## Highlights

• In this case report, this patient underwent esophagectomy, total resection of the gastric remnant, and colon reconstruction, and it is likely that methicillin-resistant *Staphylococcus aureus* (MRSA) from the upper airway system, which is not exposed to gastric acid, proliferated in the interposed colon and resulted in MRSA enteritis.
• Vancomycin-resistant *Staphylococcus aureus* (VRSA) arises from MRSA through the transfer of vancomycin resistant genes on the vanA operon to *S. aureus.*
• This case report describes the potential for rifampicin to be used as an effective treatment choice for postoperative VRSA enteritis.

## 1 Introduction

*Staphylococcus aureus* resistant to many antibiotics were isolated from several sources in the 1980s in Japan, and postoperative methicillin-resistant *Staphylococcus aureus* (MRSA) enteritis has been prevalent since 1983 with a reported mortality of approximately 10% [1]. Vancomycin is one of the most widely used antibiotics for the treatment of serious infectious caused by MRSA. However, reduced susceptibility of *S. aureus* to vancomycin has been observed in recent years. In this case report, we describe the challenges in treating a patient with vancomycin-resistant MRSA enteritis after total resection of the gastric remnant, extended lymph node dissection, and colon reconstruction. However, we successfully treated the intractable VRSA using combination therapy of vancomycin and rifampincin.

This work has been reported in line with the SCARE criteria [2].

## 2 Presentation of case

**Fig. 1:**
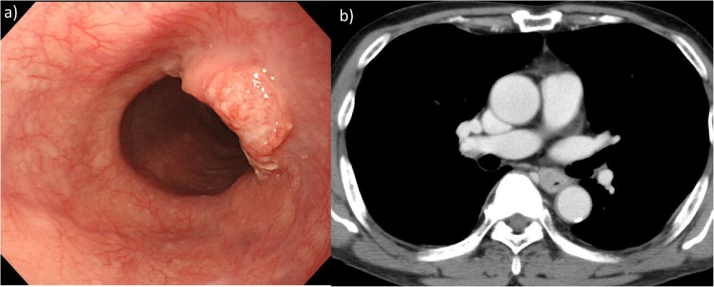
a) Endoscopic examination revealed a localized ulcerative and infiltrative tumor 27 cm from the incisors. b) The preoperative CT scan revealed no swollen lymph node.

A 66-year-old male with dysphagia was referred to our hospital for evaluation because of suspected esophageal carcinoma. He had previously undergone distal gastrectomy for a gastric ulcer at the age of 28 years. A routine preoperative throat swab culture was negative for MRSA. Endoscopy and an upper gastrointestinal series revealed a type 3 tumor on the right wall of the middle third of the esophagus (Fig. 1a). Tumor biopsy indicated moderately differentiated squamous cell carcinoma in the thoracic middle esophagus. Computed tomography scanning showed no lymph node metastasis and no tumors in other organs, such as the liver and lungs. A colonoscopy was performed, and no abnormality was found. The clinical stage of the carcinoma was T3 N0 M0, Stage IIA (Union International Cancer Control [UICC] tumor node metastasis system [TNM] classification) [3]. We initiated neoadjuvant chemotherapy, according to the Japan Clinical Oncology Group clinical practice guidelines, comprising two cycles of cisplatin plus 5-fluorouracil for a total of two courses every 3 weeks. Cisplatin was administered at a dose of 80 mg/m^2^ by 2-h intravenous drip infusion on day 1; 5-fluorouracil was administered at a dose of 800 mg/m^2^/day by continuous infusion on days 1–5. We performed right thoracotomy esophagectomy, total resection of the gastric remnant, 3-field lymph node dissection, and colon reconstruction via the retrosternal route. Surgery lasted 400 min and no complications were reported.

**Fig. 2:**
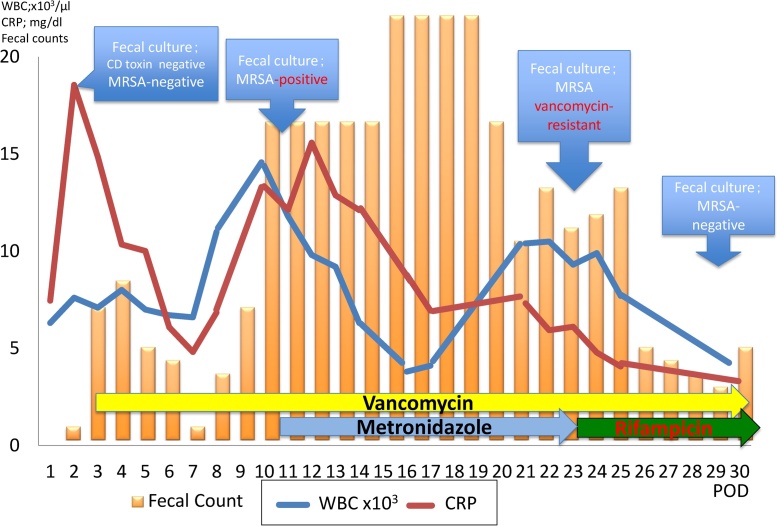
Clinical course of this case. Abbreviations: WBC; White Blood Cell, CRP; C-reactive protein, POD; Postoperative Day, MRSA; Methicillin-Resistant *Staphylococcus aureus*.

**Fig. 3:**
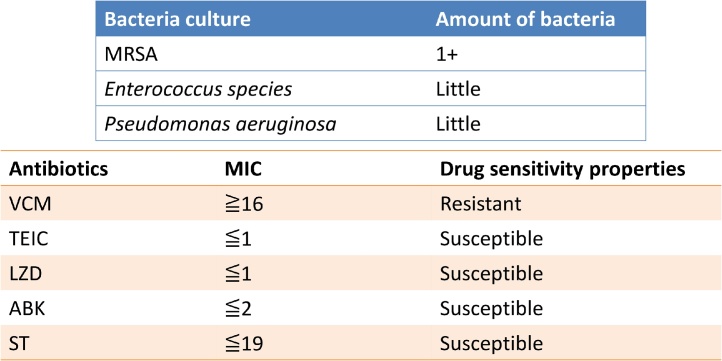
XXX.

On postoperative day (POD) 3, the patient had high fever and watery stools (Fig. 2). Serum laboratory results showed acute inflammation. Although a stool culture was negative for *Clostridium difficile* toxin and MRSA, we strongly suspected MRSA enteritis and initiated vancomycin treatment (2000 mg/4×) via feeding tube. The patient’s symptoms and laboratory data improved temporarily but worsened after POD8. The laboratory investigation revealed the white blood cell count of 14,400/mm^3^ and C-reactive protein level of 13.35 mg/dL, indicating an acute infection, and a stool culture was positive for MRSA on POD10. We added metronidazole (500 mg/1×) via feeding tube. The patient’s feces count was over 20 times per day, and his serum sodium levels and blood pressure were decreased so we administrated extracellular fluid (>3000 mL/day). Although the MRSA identified by stool culture was shown to be susceptible to vancomycin (Fig. 3), the patient’s symptoms failed to improve. On POD 24, another stool culture was performed and shown to be MRSA resistant to vancomycin. Given the subsequent diagnosis of VRSA enteritis, treatment was changed from vancomycin (2000 mg/4×) plus metronidazole (500 mg/1×) to vancomycin (2000 mg/4×) plus rifampicin (600 mg/4×) from POD 24. The patient’s symptoms and laboratory data were improved from POD 26, and a stool culture was negative for MRSA on POD 30. The patient was discharged on POD 82, and a follow-up colonoscopy 3 months after discharge showed no abnormalities. At the time of writing, the patient is alive – 5 years after surgery.

## 3 Discussion

MRSA enteritis should be considered a possible postoperative complication after gastrointestinal surgery. MRSA and *C. difficile* are important bacterial causes of postoperative enteritis. The clinical manifestations of MRSA enteritis range from mild to fatal, and some patients that receive postoperative third-generation cephem antibiotics go on to develop MRSA enteritis [4]. Postoperative MRSA enteritis is characterized by heavy, watery diarrhea, abdominal distention, and high fever, which appear on the second or third postoperative day, and oliguria, hypotension, hypoxia, and prominent leukopenia are subsequently observed. Gastrectomy is a risk factor for postoperative MRSA enteritis [5], as MRSA cannot survive in conditions below pH 4.0. MRSA in the upper respiratory system has been reported to transfer readily to feces in the low-acid stomach conditions resulting from H2 blocker therapy. In this case, this patient underwent esophagectomy, total resection of the gastric remnant, and colon reconstruction, and it is likely that MRSA from the upper airway system, which is not exposed to gastric acid, proliferated in the interposed colon and resulted in MRSA enteritis [6,7].

The antibiotics commonly used to treat MRSA are arbekacin (ABK), vancomycin (VCM), linezolid (LZD), and daptmycin (DAP) [8], although MRSA appears to have reduced susceptibility to VCM. VCM minimum inhibitory concentration (MIC) for MRSA strains has been shown to increase over time, referred to as ‘MIC creep’ [9]. Although vancomycin has been used as the standard therapy for MRSA infections to date, *Staphylococcus* isolates with decreased susceptibility to VCM (i.e., VRSA) have been reported.

VRSA is a rare, multidrug-resistant bacterium which first emerged in 2002 [10]. VRSA arises from MRSA through the transfer of vancomycin resistant genes on the vanA operon to *S. aureus* [11]. VRSA is defined as *S. aureus* with vancomycin MIC above 16 μg/mL. When vancomycin therapy is unsuccessful, several antibiotic combination therapies have the potential to be effective [12], although there is no standard therapy for VRSA described at present.

In this case report, we describe the effectiveness of a combination of vancomycin and rifampincin for VRSA enteritis. The susceptibility of the MRSA biofilm to vancomycin and rifampincin was enormous in-vivo investigation [13]. Salem et al. previously reported that rifampicin demonstrated higher efficacy than vancomycin against the MRSA biofilm [13]. This may be attributed to rifampicin’s lower molecular weight and less complex structure, which enables higher penetration through the biofilm matrix compared with vancomycin. Studies using combination treatment of vancomycin and rifampicin revealed antagonism at all concentrations with an interaction index as appreciably higher than 1, indicating strong antagonism between the two agents against the MRSA biofilm [14].

## 4 Conclusions

In our case, the patient had a high risk for MRSA enteritis because of his esophagectomy, remnant gastrectomy, and colon reconstruction. We successfully treated his postoperative VRSA enteritis by early initiation of combination therapy of vancomycin and rifampicin. This strategy may therefore represent an effective treatment choice for postoperative VRSA enteritis.

## Conflict of interest

All authors have no conflict of interest about this study.

## Funding

This research did not receive any specific grant from funding agencies in the public, commercial, or not-for-profit sectors.

## Ethical approval

This study was approved by the Ethics Committee of National Cancer Centre Hospital East. All of the participants provided informed consent and signed a human subject institutional review board consent form.

## Consent

We obtained consent to publish this case presentation from this patient.

## Author contribution

Case report concept and design acquisition of data, analysis and interpretation of data; NO, DH, drafting of the manuscript; NO, HD, critical revision of the manuscript for important intellectual content; JK, administrative, technical, or material support TF, JK, AS, YH, TS, HF, HD.

## Registration of research studies

N/A.

## Guarantor

Hiroyuki Daiko.

## Provenance and peer review

Not commissioned, externally peer-reviewed.
